# BDNF Induced Translation of Limk1 in Developing Neurons Regulates Dendrite Growth by Fine-Tuning Cofilin1 Activity

**DOI:** 10.3389/fnmol.2019.00064

**Published:** 2019-03-20

**Authors:** Sreenath Ravindran, Vijayalaxmi C. Nalavadi, Ravi S. Muddashetty

**Affiliations:** ^1^Center for Brain Development and Repair (CBDR), Institute for Stem Cell Biology and Regenerative Medicine (inStem), Bangalore, India; ^2^Manipal Academy of Higher Education, Manipal, India

**Keywords:** BDNF, cofilin1, dendrite growth, early neurodevelopment, Limk1, translation

## Abstract

Dendritic growth and branching are highly regulated processes and are essential for establishing proper neuronal connectivity. There is a critical phase of early dendrite development when these are heavily regulated by external cues such as trophic factors. Brain-derived neurotrophic factor (BDNF) is a major trophic factor known to enhance dendrite growth in cortical neurons, but the molecular underpinnings of this response are not completely understood. We have identified that BDNF induced translational regulation is an important mechanism governing dendrite development in cultured rat cortical neurons. We show that BDNF treatment for 1 h in young neurons leads to translational up-regulation of an important actin regulatory protein LIM domain kinase 1 (Limk1), increasing its level locally in the dendrites. Limk1 is a member of serine/threonine (Ser/Thr) family kinases downstream of the Rho-GTPase pathway. BDNF induced increase in Limk1 levels leads to increased phosphorylation of its target protein cofilin1. We observed that these changes are maintained for long durations of up to 48 h and are mediating increase in number of primary dendrites and total dendrite length. Thus, we show that BDNF induced protein synthesis leads to fine-tuning of the actin cytoskeletal reassembly and thereby mediate dendrite development.

## Introduction

During neuronal development, dendritic growth and patterning lay down the basic architecture of the neuronal network. This important phase is regulated by various cell-intrinsic and external cues (Scott and Luo, 2001). The temporal profile of dendrite development *in vivo* typically is non-linear (Wu et al., 1999). In the early phase of perinatal development, the dendrite branches are highly dynamic and are affected significantly by different cues. This dynamic phase of development is a critical time window which later is replaced with a stable phase where dendritic branches show minimal growth and pruning. This developmental profile is recapitulated in *in vitro* systems as well. Dendrites of cultured neurons have an initial slow phase (which also shows fast axonal growth), followed by an active phase of dendritic growth and pruning, and then a late phase of slow growth and pruning (Dotti et al., 1988). Although a large number of studies have focussed on understanding spine formation, pruning and plasticity in mature dendrites, the molecular details governing early dendrite development is not completely understood. This understanding is imperative in the context of several neurodevelopmental disorders, as defects in this critical window lead to long term and irreversible changes in the neuronal connectivity.

Similar to axons, dendrite growth and spine development also require extensive cytoskeletal re-arrangements involving both actin and microtubule filaments (Ferreira et al., 2010; Ohtani et al., 2014). Actin network, being peripherally present in the filopodia, responds to several external cues, initiating the reassembly (Scott and Luo, 2001; Da Silva and Dotti, 2002). The microtubule cytoskeleton is involved in the stabilization of the new branches initiated due to actin reassembly (Zhou et al., 2002; Hu et al., 2008; Gu and Zheng, 2009). External cues activated signaling cascades converge on these cytoskeletal elements bringing about dendrite growth (Whitford et al., 2002). Brain-derived neurotrophic factor (BDNF), a member of the neurotrophin family of proteins promotes neuronal survival and dendritic growth in the cerebral cortex and hippocampus (McAllister et al., 1995; Labelle and Leclerc, 2000; Horch and Katz, 2002). BDNF-TrkB signaling is also critical for dendritic spine enlargement and maintenance of LTP, mediated partly through mTOR dependent activation of protein synthesis (Schratt et al., 2004, 2006; Kuipers et al., 2016). Dendritic spines are actin-rich structures and spine dynamics are driven mainly by actin remodeling, thus sharing several molecular pathways with dendrite growth. Reports have shown that BDNF induced changes in spine morphology, as well as trophic factor responses in growing axons, are mediated *via* translational regulation of actin modulator proteins (Leung et al., 2006; Schratt et al., 2006; Spillane et al., 2012). These studies clearly indicate that trophic factors affect the translational profile of actin modulator proteins in neuronal compartments involving structural alterations.

Microarray-based studies have identified that translation of an actin modulator protein LIM domain kinase 1 (Limk1) is enhanced on BDNF treatment in mature as well as immature rat cortical neuronal cultures (Schratt et al., 2004). We were interested in understanding the role of BDNF mediated Limk1 translation in young neurons during the critical period of dendritic growth, and its physiological role in dendrite development. Cultured neurons are a good model system as the neurite growth profile is well characterized (Kaech and Banker, 2006) and the system is amenable to both long term and short term drug treatments. Our results show that BDNF causes translational up-regulation of Limk1 and increases its level in the dendrites. This effect persists for long period and enhances dendrite growth *via* modulating the activity of the actin-binding protein cofilin1.

## Materials and Methods

### Ethics Statement

All animal work was done with due approval from the Institutional Animal Ethics committee (IAEC) constituting Prof. Sumantra Chattarji as the chairperson and Dr. P. Krishnamurty as the CPCSEA nominee (external member) and the Institutional Biosafety Committee (IBSC), InStem, Bangalore, India.

### Primary Neuronal Culture

Primary neuronal culture was prepared from cerebral cortices of E18 rats (Sprague-Dawley) according to an established protocol (Kaech and Banker, 2006). For biochemical studies, high-density neuronal cultures (~38,000 cells per square cm) were plated on poly-L-lysine (0.2 mg/ml in borate buffer, pH 8.5) coated dishes. For the immunostaining experiments, dissociated cells were plated at lower density (~2,500 cells per square cm) on poly-L-lysine coated coverslips. Neurons were attached to the substrate in minimal essential medium with 10% fetal bovine serum (FBS, Sigma F2442) for 3 h, and coverslips were inverted onto 6-well plates containing astroglia, and grown in defined Neurobasal Medium (Invitrogen, Carlsbad, CA, USA) with GlutaMAX™ supplement (Gibco™) and B-27 supplements (Invitrogen, Carlsbad, CA, USA). Neurons were cultured for 5 days at 37°C in a 5% CO_2_ environment.

### Polysome Profiling

Polysome assay was done from cell lysate as described previously (Muddashetty et al., 2007). In brief, cell lysate was separated on 15%–45% linear sucrose gradient in presence of cycloheximide (CHX) by centrifugation at 39,000 rpm in SW41 rotor for 90 min. The sample was fractionated in 11 1.0 mL fractions with continuous UV absorbance measurement (A254). Fractions were further analyzed by western blots. Fractions were pooled (1–7 and 8–11) according to puromycin sensitivity, as assessed by UV absorbance profile and the distribution of ribosomal protein RPLP0 in western blots. Total RNA isolated from the pooled fractions using Trizol LS method according to the company protocol. Quantitative PCRs (qPCRs) performed with primers for *Limk1, β-Actin, cofilin1, Arc, Arpc3*, from the two pools.

### Quantitative PCR and Primers

**Table:** 

	Forward	Reverse
*Limk1*	GTAACCCCTACTGGATGGCG	AGTTTGGTGGACAGTAGCGG
*Arc*	AAGTTCAAGCGCTTTCTGCG	GACTCGCTGGTAAGAGCAGG
*Arpc3*	GAGACCGGACTGAGGCTTTG	CACCTCAATGCGATGCTGAC
*cofilin1*	GGCTCTGTTCTTCTGTAGCTCT	CACTGCCTTCTTGCGTTTCTT
*β-actin*	GGCTCCTAGCACCATGAAGAT	AAACGCAGCTCAGTAACAGTC

Arbitrary copy numbers calculated from a standard curve drawn from Ct values obtained from serial dilutions of cDNA.

### Immunostaining

Rat primary cortical neurons were stimulated at days *in vitro* (DIV) 5 with 50 ng/ml BDNF for 1 h. Cells were fixed with 4% PFA and processed for imaging as described before (Muddashetty et al., 2011). In brief, cells were permeabilized using TBS_50_T (0.3%) [50 mM Tris-Cl (pH 7.4) + 150 mM NaCl + 0.3% TritonX-100]. This was followed by treatment with Tris-Glycine solution (0.5 M Tris and 0.2 M Glycine) for background reduction before blocking with blocking buffer [TBS_50_T (0.1%) + 2% BSA + 2% FBS]. Primary antibodies were incubated in TBS_50_T (0.1%) + 1% BSA overnight at 4°C which was followed by washes. Alexa Fluor 488 coupled anti-mouse and Alexa Fluor 555 coupled anti-rabbit secondary antibodies were incubated for 1 h at room temperature. Finally, coverslips were mounted for imaging using Mowiol^®^ 4-88 mounting media. Images were acquired on FV3000 confocal microscope (Olympus) using Plan Apo 60×, NA 1.42, oil immersion objective. Z-series of 6–10 stacks with XY sampling density of 0.094 μm/pixel were taken at 0.5 micron step size from all dendrites. Imaging conditions were kept constant across experiments. Antibodies against p-ser3-cofilin1 (ab12866), p-thr508-Limk1 (ab38508) and total Limk1 (ab119084) were purchased from Abcam. Total cofilin1 (WH0001072M4) and alpha-tubulin (T9026) from Sigma.

### Neurite Growth Assays

Rat primary cortical neurons were stimulated at DIV 5 with 50 ng/ml BDNF for 48 h. Cells were fixed with 4% PFA and immunostained for microtubule-associated protein2 (Map2) and Microtubule-associated protein Tau (Tau) proteins with respective antibodies (Map2-Sigma M9942, Tau- Abcam ab76128). Dendrites (Map2 positive and Tau negative) were traced using the NeuronJ plugin in ImageJ software as described (Meijering et al., 2004). The threshold was set up manually for the images and Sholl analysis was carried out using the sholl plugin in ImageJ (Ferreira et al., 2014) with 10 micron steps. For Limk1 knockdown experiments, DIV 5 cortical neurons were transfected with scrambled or Limk1 siRNA (Thermo Fisher, Waltham, MA, USA, s134717) along with EGFP, followed by BDNF treatment for 48 h. Dendrites were identified based on their short, distally tapering branches from the cell body and traced using NeuronStudio software (CNIC, v 0.9.92; Wearne et al., 2005). All the analysis were done with experimenter blind to the condition.

### Live Imaging to Quantitate Dendrite Growth

Rat primary cortical neurons were cultured on Nunc glass bottom Petri dishes at a density of 200,000 cells per dish. Cells were transfected at DIV 4 using Lifeact-mCherry (Addgene, Watertown, MA, USA) vector by magnetofection as per the company protocol. Images were acquired on FV3000 confocal microscope (Olympus) using Plan Apo 60×, NA 1.42, oil immersion objective, with 37°C temperature and 5% CO_2_ maintenance. Dendrites were identified as short, distally tapering branches from the cell body. Images were captured as Z-series of 6–10 stacks with XY sampling density of 0.090 μm/pixel at 0.5 micron step size from all dendrites at 5 min interval using a high sensitivity PMT detector. Cells were imaged for a period of 1 h after which 50 ng/ml BDNF was added and imaging was continued for one more hour. For analysis, the Z stacks were compressed by maximum intensity projection using ImageJ and imported into Imaris software (Bitplane AG, x64, v 9.0.1). The FilamentTracer tool in Imaris was used for automatic tracing of dendrites and identification of dendrite tips. Tip trajectories were traced and quantified for the entire imaging duration.

### Fixed Cell F-Actin Measurement

F-actin visualization, Alexa Fluor 488 phalloidin (Invitrogen, Carlsbad, CA, USA) was added to the secondary antibody solution (1:50 dilution) during immunostaining and incubated for 1 h. F-actin levels were quantified as an intensity ratio of phalloidin to Map2 fluorescence. As an alternate method, rat primary cortical neurons were transfected at DIV 4 with equimolar concentrations of Lifeact-mCherry and EGFP C1 vectors and treated at DIV 5 with 50 ng/ml for 48 h. Cells were fixed at DIV 7 and F-actin measured as mean intensity ratio of mCherry to EGFP.

### Statistical Analysis

Statistical significance was calculated using unpaired Student’s *t*-test for biochemical experiments. For immunostaining based quantifications, Mann Whitney test was used. Data plotted as mean ± SEM unless described otherwise. Value of *p* < 0.05 was considered statistically significant.

## Results

### BDNF Activates Limk1 Translation in Immature Neurons in Culture

**Figure 1:**
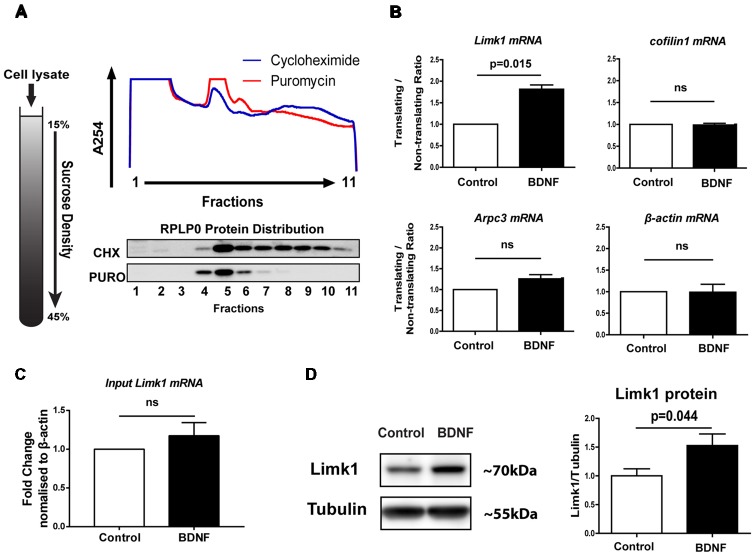
Brain-derived neurotrophic factor (BDNF) increases LIM domain kinase (Limk1) synthesis in days *in vitro* (DIV) 5 cortical neurons through translational up regulation. **(A)** Graphic showing 15%–45% linear sucrose gradient (Left) and representative A254 profile from polysome fractionation of 5 DIV cortical neurons, treated with cycloheximide (CHX) or puromycin (Right Top). Right bottom panel shows representative immunoblots for RPLP0 from polysome profiling fractions. **(B)** Quantification of mRNA distribution from DIV 5 cortical neurons treated with 50 ng/ml BDNF for 20 min after polysome profiling. Data plotted as a ratio of copy numbers in translating to non-translating fractions normalized to control levels (*n* = 3, Welch’s *t*-test, mean ± SEM). **(C)** Quantification of Limk1 mRNA levels from DIV 5 cortical neurons treated with 50 ng/ml BDNF for 20 min. (*n* = 4, Welch’s *t*-test, mean ± SEM). **(D)** Representative immunoblots and quantification for Limk1 protein from DIV 5 cortical neurons treated with 50 ng/ml BDNF for 1 h (*n* = 7, Unpaired Student’s *t*-test, mean ± SEM); ns, not significant.

We used primary neurons cultured from embryonic (E18) rat cerebral cortex for this study. We have conducted our experiments on DIV-5 neurons which are in their stage four growth phase and do not possess mature synapses (Dotti et al., 1988; Kaech and Banker, 2006). To study the influence of BDNF on translation of actin modulators in these young neurons, we used the translational assay of polysome profiling (Schratt et al., 2004; Muddashetty et al., 2007). In this assay, post-nuclear cell lysates were subjected to ultracentrifugation on a linear sucrose gradient (Figure 1A). Fractions were collected with measurement of UV absorbance at 254 nm to identify various ribosomal pools. UV absorbance profile, as well as ribosomal protein RPLP0 distribution in puromycin vs. CHX treated cells, were used to identify the fractions containing actively translating polysomes (Figure 1A). Based on puromycin sensitivity, fractions were pooled into translating (8–11) and non-translating (1–7) pools. DIV 5 cortical neurons were treated with 50 ng/ml BDNF for 20 min and distribution of selected mRNAs in these pools were assayed by qPCR (Figure 1B). We screened for several candidates previously reported as actin cytoskeletal modulators in mature synapses or growing axons (Leung et al., 2006; Messaoudi et al., 2007; Spillane et al., 2012). Among the candidates, *Limk1* mRNA showed a significant increase in polysomal distribution on BDNF treatment compared to control; suggesting that translation of* Limk1* mRNA is up-regulated on BDNF treatment (Figure 1B). Other mRNAs that we examined include *β-actin, cofilin1, and Arpc*3 which did not show significant change in their polysomal distribution (Figure 1B). To measure the transcriptional contribution, we examined the total levels of *Limk1* mRNA on BDNF treatment. We did not observe a significant increase in total Limk1 mRNA levels as quantitated by qPCR. (Figure 1C). To validate the change in translation at the protein level, we quantified Limk1 levels by immunoblotting and found that 1 h of BDNF treatment resulted in 53 ± 20% increase in Limk1 levels compared to control (Figure 1D). Thus, the change in mRNA translational profile for Limk1 is reflected in the protein levels. Both polysome profiling and immunoblotting results confirm that BDNF causes translational up-regulation of Limk1 in immature neurons. Next, we studied whether this newly synthesized protein is functionally active.

### BDNF Induces Phosphorylation of Cofilin1 in a Translation-Dependent Manner

**Figure 2:**
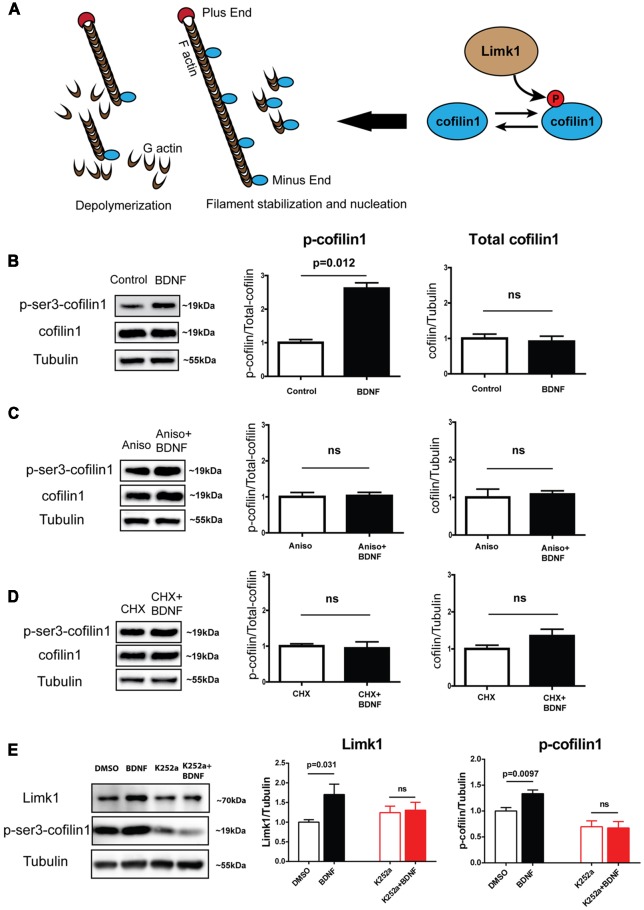
BDNF leads to increase in phosphorylation of cofilin1 in a protein synthesis-dependent manner. **(A)** Schematic showing Limk1 and cofilin1 function. **(B)** Representative immunoblots (Left) and quantification of phosphorylated (Center) and total cofilin (Right) from DIV 5 cortical neurons with BDNF treatment for 1 h. **(C)** Representative immunoblots (Left) and quantification of phosphorylated (Center) and total cofilin (Right) from DIV 5 cortical neurons with BDNF treatment for 1 h in the presence of 40 μM anisomycin. **(D)** Representative immunoblots (Left) and quantification of phosphorylated (Center) and total cofilin (Right) from DIV 5 cortical neurons with BDNF treatment for 1 h in the presence of 100 μM CHX. **(E)** Representative immunoblots (Left) and quantification of Limk1 (Center) and phosphorylated cofilin (Right) from DIV 5 cortical neurons with BDNF treatment for 1 h in the presence of 200 nM K252a (*n* = 3–5, Unpaired Student’s *t*-test, mean ± SEM), ns, not significant.

Limk1 is a serine/threonine (Ser/Thr) kinase downstream of Rho-GTPase signaling pathway that plays a key role in actin filament dynamics. Phosphorylated Limk1 (active form) phosphorylates the actin-binding protein cofilin1 at Ser-3 (Arber et al., 1998; Yang et al., 1998; Figure 2A). Cofilin1 is a member of ADF/cofilin family of proteins. As shown in the illustration (Figure 2A), cofilin1 has multiple effects on actin polymerization depending on the stoichiometry of binding. At lower concentration, it binds to F-actin and leads to depolymerization as well as filament breaks. At higher concentrations, it can stabilize F-actin and cause nucleation (Andrianantoandro and Pollard, 2006; Van Troys et al., 2008). Phosphorylated cofilin1 loses its actin binding ability and becomes inactive. As both Limk1 and cofilin1 are important for neurite growth (Meberg and Bamburg, 2000; Endo et al., 2003, 2007; Lee-Hoeflich et al., 2004), we looked at the phosphorylation status of cofilin1, a Limk1 target protein following BDNF treatment. We found that BDNF induces a robust increase in phosphorylation of cofilin1 in DIV 5 neurons (Figure 2B) with no change in the total cofilin1 levels (Figure 2B). This is in accordance with the translational profiling which showed that translation of cofilin1 mRNA was unaffected on BDNF treatment (Figure 1B). The change in phosphorylation of cofilin1 was abrogated by blocking new protein synthesis using CHX or anisomycin (Figures 2C,D). This indicates that BDNF induced cofilin1 phosphorylation is dependent on BDNF induced translation. To test if these changes are dependent on TrkB activation, we used the tyrosine protein kinase inhibitor K252a to block TrkB phosphorylation. Both BDNF mediated Limk1 synthesis, as well as cofilin1 phosphorylation was completely blocked by inhibiting TrkB activation (Figure 2E). These results suggest that BDNF induced TrkB phosphorylation is required for Limk1 synthesis and cofilin1 phosphorylation. However, we did not any change in Limk1 and p-cofilin1 levels on treatment with the second TrkB ligand Neurotrophin-4 (NT4) (Supplementary Figure S1), indicating the specificity of this response. Our data so far shows that similar to its role in dendritic spines (Schratt et al., 2006), BDNF is involved in translational fine-tuning of some key actin modulators in immature neurons. Since the majority of the cell volume is contributed by the somatodendritic compartment, this implies that dendrite development is regulated by this process. Considering that BDNF is a well-known factor regulating dendrite growth and that actin cytoskeletal changes are indispensable in mediating neurite growth, we tested whether BDNF induced Limk1 synthesis and consequent cofilin phosphorylation is important for dendrite growth.

### BDNF Leads to Increase in Limk1 Levels and Phosphorylation of Cofilin1 in the Dendrites

**Figure 3:**
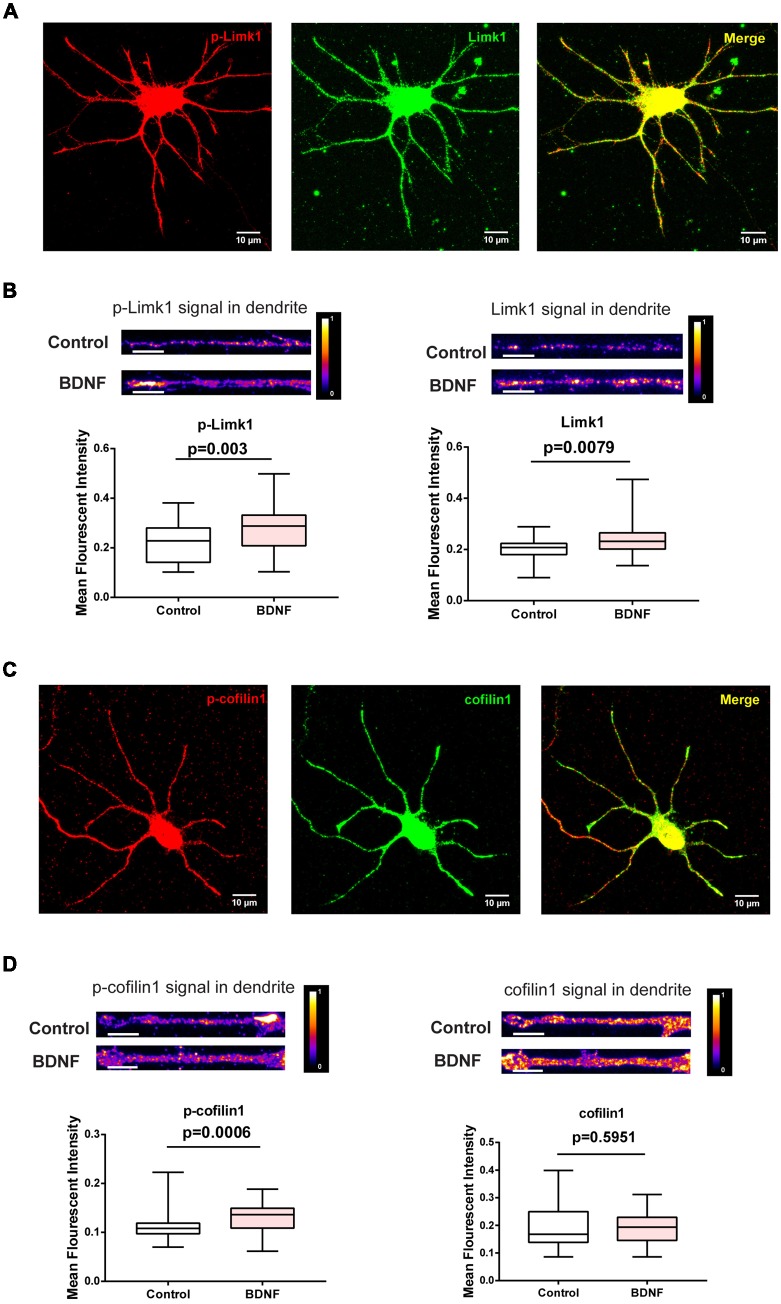
BDNF leads to increased dendritic Limk1 levels and phosphorylation of cofilin1. **(A)** Immunofluorescence images showing phosphorylated (Red) and total Limk1 (Green) distribution in the dendrites of DIV 5 cortical neurons. **(B)** Representative intensity profiles (Top) and quantification (Bottom) of mean fluorescent intensities from the entire dendrite for p-Limk1 (Left) and Limk1 (Right) from DIV 5 cortical neurons treated with 50 ng/ml BDNF for 1 h (*n* = 38–39 neurons from three independent experiments). **(C)** Immunofluorescence images showing phosphorylated (Red) and total cofilin1 (Green) distribution in the dendrites of DIV 5 cortical neurons. **(D)** Representative intensity profiles (Top) and quantification (Bottom) of mean fluorescent intensities from the entire dendrite for p-cofiin1 (Left) and cofilin1 (Right) from DIV 5 cortical neurons treated with 50 ng/ml BDNF for 1 h (*n* = 34–46 neurons from three independent experiments). Box and whisker plots show median, first and third quartiles with error bars representing the minimum and maximum data points (Mann-Whitney test).

**Figure 4:**
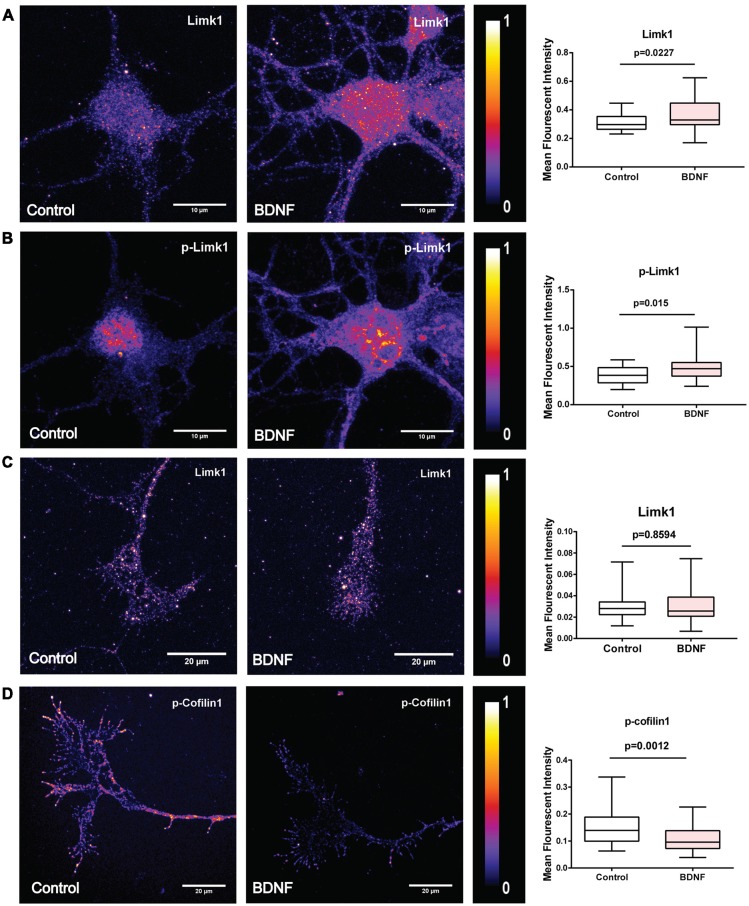
BDNF leads to increased Limk1 levels in the cell body. In the axonal growth cones BDNF did not affect Limk1 levels, but reduced phosphorylation of cofilin1. **(A)** Representative heat map images of control (Left) and BDNF treated (Center) cortical neurons immunostained for Limk1. Right panel shows the quantification of the signal from the cell body. **(B)** Representative heat map images of control (Left) and BDNF treated (Center) cortical neurons immunostained for p-Limk1. Right panel shows the quantification of the signal from the cell body. **(C)** Representative heat map images of axonal growth-cones from control (Left) and BDNF treated (Center) cortical neurons immunostained for Limk1. Right panel shows the quantification of the signal. **(D)** Representative heat map images of axonal growth-cones from control (Left) and BDNF treated (Center) cortical neurons immunostained for p-cofilin1. Right panel shows the quantification of the signal (*n* = 30–36 neurons from three independent experiments. Box and whisker plots show median, first and third quartiles with error bars representing the minimum and maximum data points. Mann-Whitney test).

To check if BDNF affects Limk1 levels locally in dendrites, we measured Limk1 levels in the dendrites of DIV 5 neurons by quantitative immunofluorescence. Both total and phosphorylated Limk1 are present in the cell body as well as throughout the length of the dendrite (Figure 3A). We observed a significant increase in total as well as phosphorylated Limk1 levels in the whole dendrites following 1 h of BDNF treatment (Figure 3B). Next, we checked the levels of phosphorylation of cofilin1 in the dendrites. Similar to Limk1 distribution, both total and phosphorylated cofilin1 is present throughout dendrites (Figure 3C). Corresponding to the increase in Limk1 levels, the phosphorylated cofilin1 levels were increased in the dendrites in response to BDNF treatment (Figure 3D). Similar to the immunoblot data, total cofilin1 levels were unaffected by BDNF treatment (Figure 3D). We also found that total and phosphorylated Limk1 levels in the cell body increased on BDNF treatment (Figures 4A,B) showing that this is a somatodendritic effect. Contrary to the dendritic increase of Limk1, there was no change in Limk1 levels in the axonal growth cones (Figure 4C). Similarly, the phosphorylation of cofilin1 on BDNF treatment was not observed in the axonal compartment. As reported previously (Marsick et al., 2010) with positive trophic factors, we observed a reduction in cofilin1 phosphorylation on BDNF treatment in axonal growth cones (Figure 4D) while proximal axonal segments did not show a significant change (data not shown). Thus, our data show that BDNF mediated rise in Limk1 levels is present in the cell body and dendrites, which mediates the phosphorylation of cofilin1. This translational response is not observed in the axons. To test whether these molecular changes are present in the later stages of dendrite development, we quantified dendritic levels of Limk1 and p-cofilin1 in DIV10 neurons following BDNF treatment by quantitative immunofluorescence. Similar to DIV5 data, we observed a significant increase in Limk1 as well as phosphorylated cofilin1 levels in the DIV10 dendrites following 1 h of BDNF treatment (Supplementary Figure S2), showing that these responses are preserved in later stages of dendritic growth as well.

### BDNF-Induced Limk1 Synthesis Is Sustained for 48 h and Mediate Dendrite Growth

**Figure 5:**
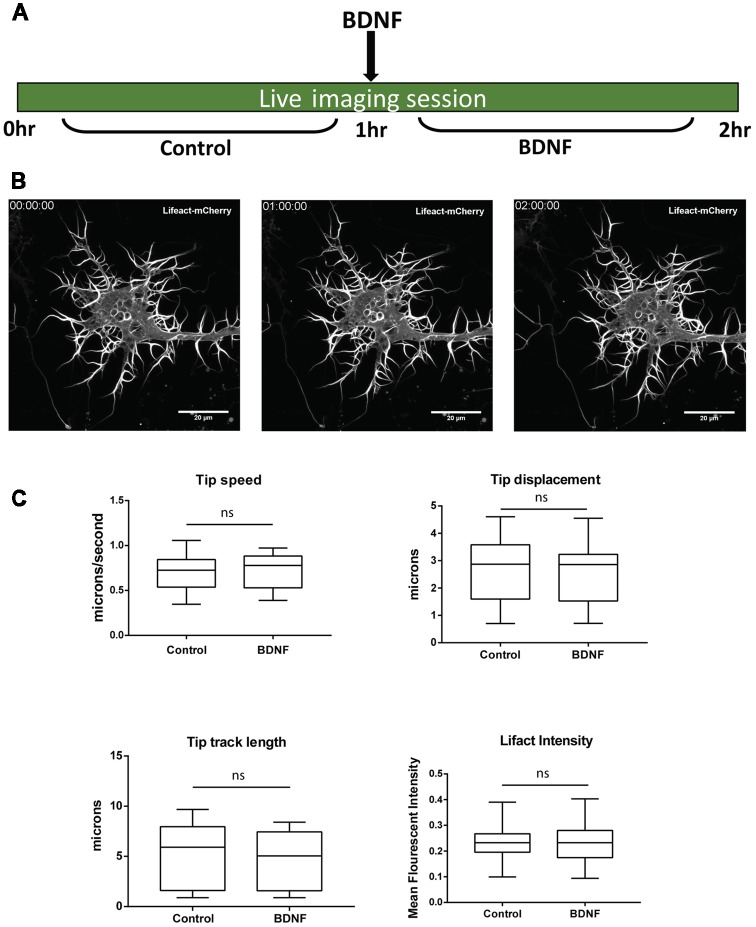
One hour BDNF treatment did not lead to detectable changes in dendrite growth visualized by live imaging. **(A)** Schematic showing live imaging paradigm. Cortical neurons are transfected with Lifeact-mCherry at DIV 4 and imaged at DIV 5 for 1 h before and after BDNF addition. **(B)** Representative images from Lifeact-mCherry transfected cortical neurons at time points of 0, 1 and 2 h as indicated. **(C)** Quantification of dendrite tip speed (Top Left), displacement (Top Right), track length (Bottom Left) and Lifeact intensity in dendritic filopodia (Bottom Right) from control and BDNF treated conditions as described in **(A,B)** (*n* = 14 neurons from three independent experiments. Box and whisker plots show median, first and third quartiles with error bars representing minimum and maximum data points. Mann-Whitney test), ns, not significant.

Next, we investigated whether newly synthesized Limk1 is important for mediating dendrite growth induced by BDNF. We studied the physiological response after 1 h of BDNF treatment, which brings about some key actin modulator protein changes. We standardized a short-term live imaging assay to visualize dendrite growth through labeling filamentous actin with mCherry tagged Lifeact. Cortical neurons were transfected by Lifeact-mCherry at DIV 4 and imaged at DIV 5. Growing dendritic filopodia were identified based on their morphology and imaged for a period of 1 h before and after BDNF addition (Figure 5A). Using this method we observed growth and retractions in the dendrite tips during this period (Figure 5B). The filopodial length and tip dynamics were tracked using FilamentTracer in Imaris software and was quantitated as parameters of tip track length, displacement and speed (Figure 5C) before and after BDNF addition. The F-actin content was also quantified by measuring Lifeact intensity (Figure 5C). Dendritic filopodia showed a high rate of dynamicity during the imaging period, but we did not find the difference statistically significant for the measured parameters between control and BDNF treated neurons. This points out that the BDNF induces morphological changes in dendrites could not be captured at shorter time scales but needed longer periods.

**Figure 6:**
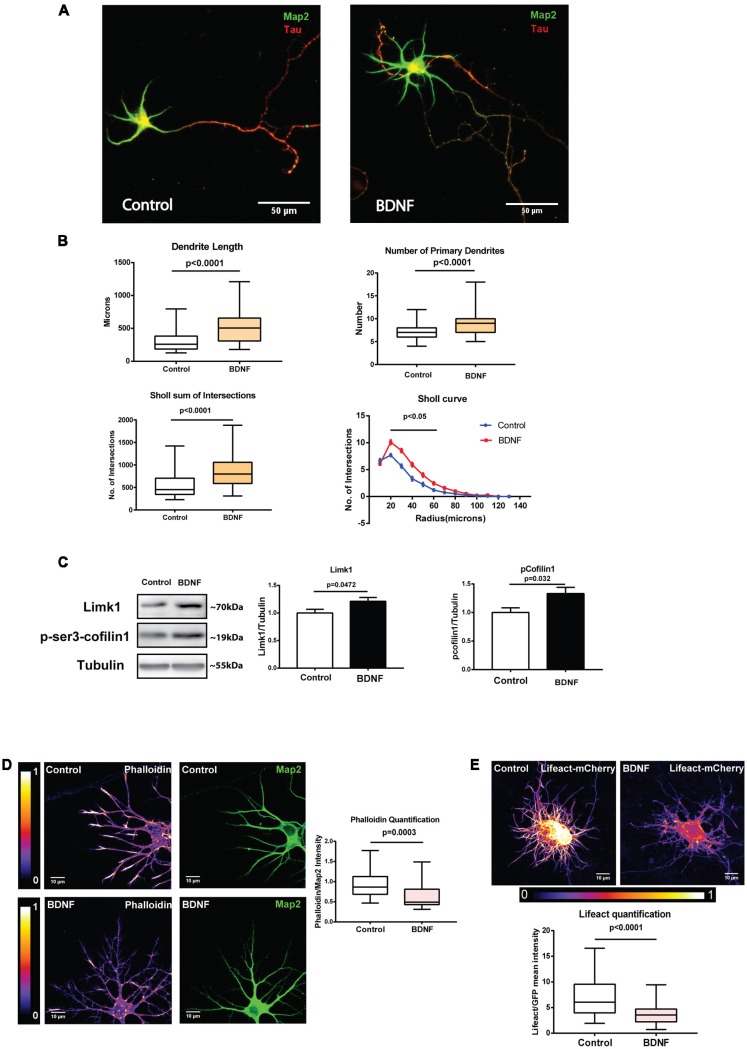
BDNF-induced Limk1 synthesis is sustained for 48 h and is associated with dissolution of F-actin and dendritic growth. **(A)** Representative images of neurons treated with or without 50 ng/ml BDNF for 48 h, fixed and immunostained for microtubule-associated protein2 (Map2; Green) and Tau (Red). **(B)** Quantification of total dendritic length (Top Left), number of primary dendrites (Top Right) total intersections by Sholl analysis (Bottom Left) and Sholl curves (Bottom Right) from control and BDNF treated cortical neurons from the experiment described in Figure 6A (*n* = 49–61 neurons across three independent experiments, Box and whisker plots show median, first and third quartiles with error bars representing minimum and maximum data points, Mann Whitney test). **(C)** Representative immunoblots (Left) and quantification of Limk1 (Center) and p-cofilin1 (Right) levels from control or 48 h BDNF treated cortical neurons (*n* = 7, Unpaired Student’s *t*-test, mean ± SEM). **(D)** Representative images (Left) and quantification (Center) of phalloidin from neurons treated with or without 50 ng/ml BDNF for 48 h. Mean intensity of phalloidin normalized to Map2 (*n* = 23 neurons from two independent experiments, Mann Whitney test). **(E)** Representative images (Top) and quantification of F-actin (Bottom) from neurons treated with or without 50 ng/ml BDNF for 48 h (Right) transfected with Lifeact-mCherry. Mean intensity of mCherry normalized to EGFP (*n* = 49–50 neurons from three independent experiments, Mann-Whitney test).

To confirm this, we measured total dendrite length after long term BDNF treatment. DIV 5 neurons treated with BDNF for 48 h were fixed and immunostained for Map2 to visualize the entire dendritic arbor (Figure 6A). All dendrites were traced semi-automatically using NeuronJ plugin in ImageJ software. Tau-positive axons were excluded from the analysis. As reported before (McAllister et al., 1996), this long term BDNF treatment caused a robust increase in total dendrite length and number of primary dendrites in cultured neurons compared to the controls (Figure 6B). An increase in the number of intersections was also observed in the Sholl analysis (Figure 6B). Together, the above results show that even though we can detect the molecular changes in Limk1 translation and cofilin1 phosphorylation as early as 1 h, morphological changes are detected only after a long term BDNF treatment. One possible explanation could be that these molecular changes are sustained longer to bring about detectable morphological changes. To check this possibility, we measured Limk1 and p-cofilin1 levels after 48 h of BDNF treatment by immunoblotting. In accordance with our hypothesis, we found that both Limk1 and p-cofilin levels remain elevated after 48 h of BDNF treatment (Figure 6C). Although correlative at this stage, our data suggests that sustained higher levels of Limk1 is important in mediating BDNF induced dendritic growth. To find the effect of this on actin polymerization, we measured F-actin levels by phalloidin staining as well as Lifeact quantification. In both assays, we found that 48 h BDNF treatment led to a significant reduction in F-actin levels in the dendrites (Figures 6D,E). This shows that the primary role of cofilin1 in the dendrites is either stabilization of actin filaments or actin nucleation. BDNF leads to deactivation of cofilin1 through Limk1 synthesis, thus causing a reduction in the F-actin pool.

**Figure 7:**
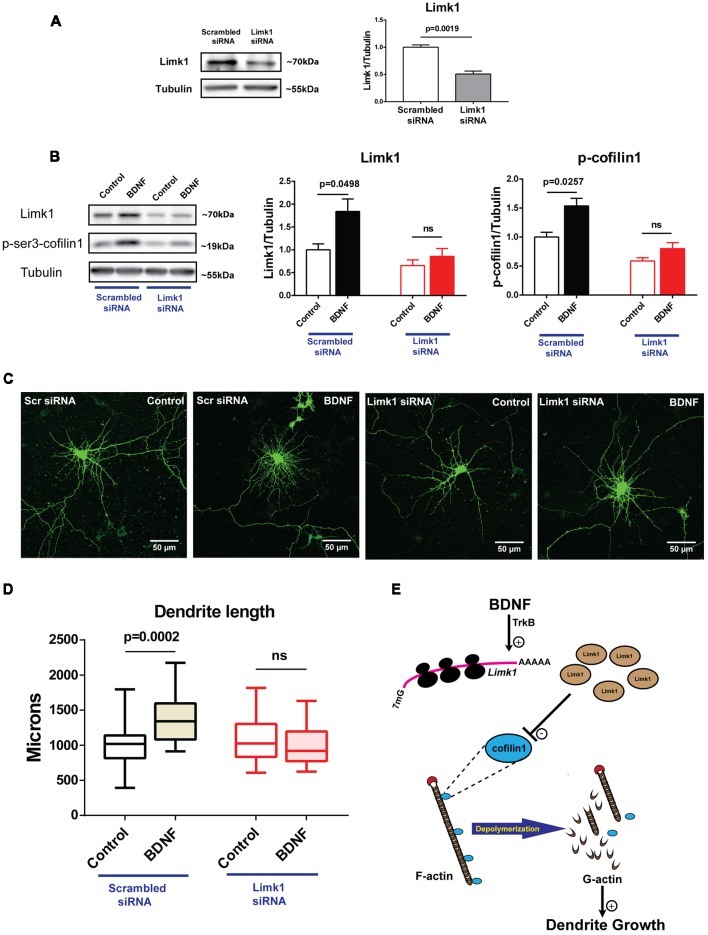
Acute knockdown of Limk1 affects BDNF mediated dendrite growth. **(A)** Representative immunoblots (Left) and quantification (Right) of Limk1 from Neuro 2a cells transfected with Limk1 or scrambled siRNA (*n* = 3, Unpaired Student’s *t*-test, mean ± SEM). **(B)** Representative immunoblots (Left) and quantification of Limk1 (Center) and p-cofilin1 (Right) from DIV5 neurons transfected with Limk1 or scrambled siRNA and treated with or without 50 ng/ml BDNF for 48 h (*n* = 3, Unpaired Student’s *t*-test, mean ± SEM). **(C)** Representative images of DIV5 neurons co-transfected with Limk1 or scrambled siRNA and EGFP and treated with or without 50 ng/ml BDNF for 48 h. **(D)** Quantification of total dendritic length from control and BDNF treated cortical neurons from the experiment described in (**C**; *n* = 23–26 neurons across two independent experiments, Box and whisker plots show median, first and third quartiles with error bars representing minimum and maximum data points, Mann Whitney test). **(E)** Model showing BDNF activated Limk1 synthesis leading to phosphorylation of cofilin1 and reduced dendritic F-actin level, thus playing important role in dendrite growth, ns, not significant.

To confirm the role of new Limk1 synthesis on BDNF mediated dendrite growth, we performed an acute Limk1 knockdown just prior to BDNF treatment. We used a specific siRNA against *Limk1* mRNA for this experiment. Limk1 knockdown was validated at the protein level compared to scrambled siRNA transfection by immunoblots (Figure 7A). To test the role for Limk1 synthesis on dendrite growth, we transfected DIV 5 cortical neurons with Limk1 or scrambled siRNA followed immediately by BDNF treatment for 48 h. Such a paradigm was used so that basal levels of Limk1 is only minimally perturbed, but any new Limk1 synthesis is inhibited. Using immunoblots, we verified that Limk1 siRNA effectively prevented BDNF mediated increased Limk1 levels as well as phosphorylation of cofilin1 (Figure 7B). This confirms that new Limk1 synthesis on BDNF stimulation is required for phosphorylation of cofilin1. For quantifying dendrite length, neurons were visualized by co-transfection of EGFP. We observed that Limk1 siRNA transfection prevented BDNF induced increase in total dendrite length, while scrambled siRNA did not have any effect (Figures 7C,D). This shows that BDNF mediated increase in Limk1 levels plays a critical role in BDNF mediated-dendrite growth.

## Discussion

Defects in activity-mediated protein synthesis are thought to be a primary cause of pathophysiology for several neurodevelopmental disorders (Kelleher and Bear, 2008; Liu-Yesucevitz et al., 2011). The role of activity mediated translation is well studied in the context of spine development and synaptic signaling (Fernandez-Moya et al., 2014). It is also established that synaptic protein synthesis is critical for long term plasticity (Kang and Schuman, 1996; Costa-Mattioli et al., 2009). But its role in dendrite morphogenesis remains largely overlooked in the field of neurodevelopment, studies are largely confined to axonal growth cones. In the current study, we focused on the role activity mediated translation regulation at this critical juncture of neuronal development which could be important in many neurodevelopmental disorders.

We demonstrate that activity-mediated translation plays an important role at the stage of robust dendritic growth and arborization. Multiple reports show that actin modulator proteins are important targets for this mode of translational regulation (Leung et al., 2006; Spillane et al., 2012; Choi et al., 2018). Our study shows this mode of regulation fine tune the local proteome of growing dendrites, thereby significantly affecting dendrite growth. We show that BDNF, a key neurotropic factor, affects dendrite growth, partially through driving translation of a Ser/Thr kinase Limk1. Exogenous BDNF stimulation in DIV5 neurons caused increased translation of Limk1 which is a key actin modulator protein. We validated this BDNF mediated translation of Limk1 mRNA by polysome profiling as well as increased Limk1 protein by immunoblot. As depicted in the model (Figure 7E), up-regulation of translation on BDNF treatment results in an increase in Limk1 in the dendrites and increased phosphorylation of its target, cofilin1. Phosphorylation of cofilin1 reduces its actin binding activity, leading to a decrease in the F-actin pool in dendrites. Interestingly we observed these specific molecular changes in dendrites and cell bodies, but not in the axons, suggesting there could be different molecular cascades activated in the axons and dendrites in response to the same trophic factor. This observation also suggests that the basal function of cofilin1 is likely to be different in axons and dendrites. Dendritic cofilin1 could be primarily mediating filament stabilization or actin nucleation. BDNF induced cofilin1 phosphorylation and subsequent dissolution of actin filaments could be important for microtubule invasion to the filopodia leading to branch stabilization and eventual increase in the dendrite length (Poulain and Sobel, 2010). These molecular change that we observed persists for durations up to 48 h and is important for mediating the physiological effect of enhanced dendritic growth.

In this study, we focussed primarily of exogenous BDNF on the pyramidal neurons in the rat cerebral cortical cultures. It remains to be studied whether other neuronal subtypes also show a similar response. We have also not explored the role of endogenous BDNF in the cultures. Further studies are required to address these questions as well as to understand the mechanism of this translational regulation. Exploring the pathways involved in such regulation would provide key insights into understanding the pathophysiology of several neurodevelopmental disorders in which dysregulation of translation is reported. Previous studies on such disorders have focussed mainly on synaptic dysfunction. Considering that both dendrite and spine development share molecular pathways pertaining to actin rearrangement, dendritic growth defects are largely overlooked in such disorders. Studying the dendritic arbor at later developmental stages might not reveal significant deficits, probably due to compensatory mechanisms. Our study indicates strongly the need to study the early developmental stages of dendritic arborization.

## Data Availability

The datasets generated for this study are available on request to the corresponding author.

## Author Contributions

SR and RM designed the experiments, analyzed the results and wrote the manuscript. SR carried out the experiments. VN optimized the primary neuronal culture and contributed to the image analysis.

## Conflict of Interest Statement

The authors declare that the research was conducted in the absence of any commercial or financial relationships that could be construed as a potential conflict of interest.
