# Supplementary material for: BDNF Induced Translation of Limk1 in Developing Neurons Regulates Dendrite Growth by Fine-Tuning Cofilin1 Activity
Source: Front Mol Neurosci. 2019 Mar 20;12:64. doi: 10.3389/fnmol.2019.00064 (PMC6436473; doi:10.3389/fnmol.2019.00064)
Supplement: Supplementary file 1 [file Image_1.pdf]

BDNF Induced Translation of Limk1 in Developing Neurons Regulates Dendrite Growth by Fine-tuning Cofilin1 Activity.

Supplementary Figure 1

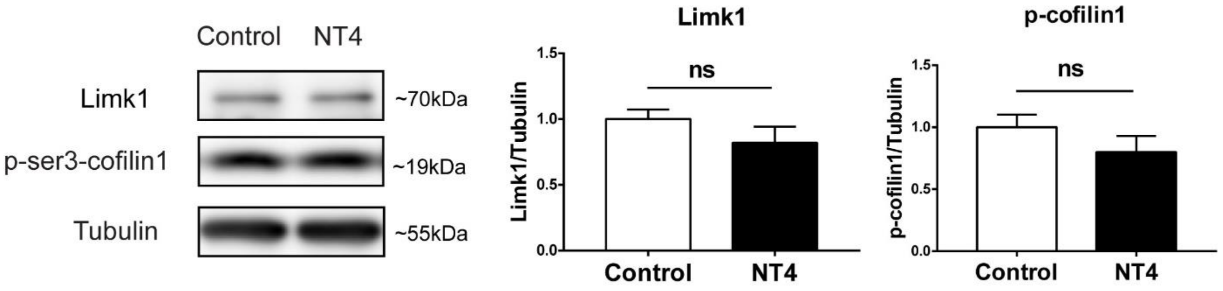

Supplementary Figure 2

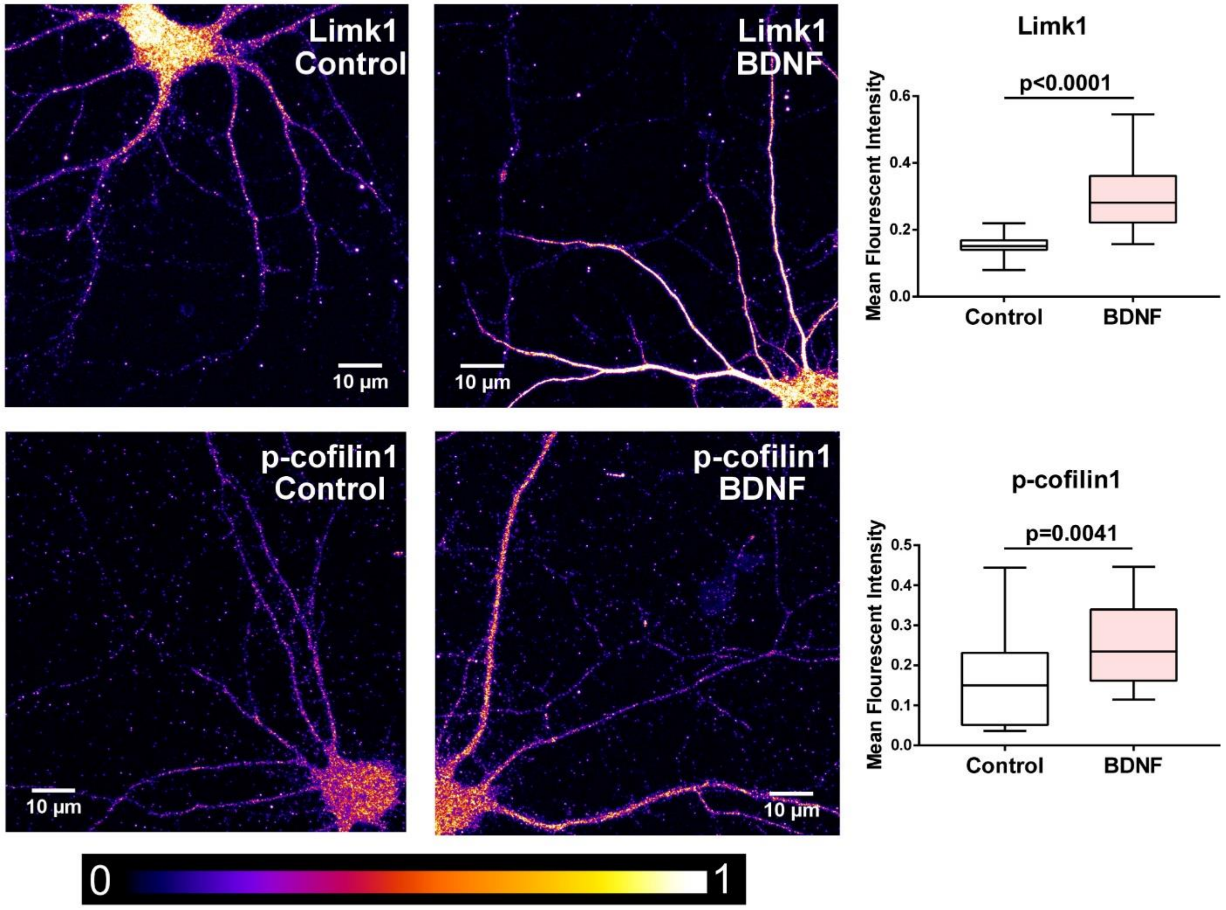

Supplementary Figure 1

NT4 treatment did not affect Limk1 levels or phosphorylation of cofilin1

A. Representative immunoblots (Left) and quantification for Limk1 (Center) and p-cofilin1 (Right) from DIV 5 cortical neurons treated with 50ng/ml NT4 for 1 hour (n=5, Unpaired Student’s t-test, mean ± s.e.m). ns-Not significant

Supplementary Figure 2

BDNF leads to increased dendritic Limk1 levels and phosphorylation of cofiin1 in DIV 10 neurons.

A. Representative intensity profiles (Left and Center) and quantification (Right) of mean fluorescent intensities from the dendrites for Limk1 (Top) and p-cofilin1 (Bottom) from DIV 10 cortical neurons treated with 50ng/ml BDNF for 1 hour (n=21-25 neurons from 2 independent experiments).

Box and whisker plots show median, first and third quartiles with error bars representing the minimum and maximum data points (Mann Whitney test).
